# Supplementary material for: Assessing the Long-Term Role of Vaccination against HPV after Loop Electrosurgical Excision Procedure (LEEP): A Propensity-Score Matched Comparison
Source: Vaccines (Basel). 2020 Dec 1;8(4):717. doi: 10.3390/vaccines8040717 (PMC7711506; doi:10.3390/vaccines8040717)
Supplement: Supplementary file 1 [file vaccines-08-00717-s001.pdf]

Table S1: Factors predicting persistence/recurrence of cervical dysplasia*.

	Univariate Analysis		Multivariate Analysis	
	**HR (95%CI)**	**P Value**	**HR (95%CI)**	**P Value**
Age, years	0.95 (0.84, 1.08)	0.456	-	-
BMI, kg/m2	0.88 (0.72, 1.08)	0.243		--
HR-HPV detected		0.171		-
No	Reference		-	
Yes	0.38 (0.10, 1.50)		-	
Type of cervical dysplasia		0.081		0.231
CIN2	Reference		Reference	
CIN3	3.15 (0.86, 11.4)		2.30 (0.58, 9.08)	
Positive margins		0.01		0.363
No	Reference		Reference	
Yes	4.32 (1.41, 13.2)		0.43 (0.07, 2.60)	
Positive esocervical margins		0.101		-
No	Reference		-	
Yes	2.94 (0.81, 10.7)		-	
Positive endocervical margins		0.028		0.513
No	Reference		Reference	
Yes	4.23 (1.16, 15.4)		2.24 (0.19, 25.2)	
Vaccination		0.185		-
No	Reference		-	
Yes	0.36 (0.08, 1.62)		-	
HPV persistence *		0.02		0.380
No	Reference		Reference	
Yes	4.47 (1.26, 15.8)		1.96 (0.43, 8.81)	

Abbreviation: BMI, body mass index; CIN, cervical intraepithelial neoplasia; HR, high-risk; HPV, human papillomavirus; HR, hazard ratio; CI, confidence intervals. *The present analysis included only patients included in the propensity-matched cohort in order to avoid the dilution effect of vaccine use.
